# Recruitment of older adult-caregiver dyads during the COVID-19 pandemic: an example from a study to evaluate a novel activities of daily living (ADL) sensor system

**DOI:** 10.3389/frdem.2023.1271754

**Published:** 2023-10-19

**Authors:** Rachel Williams, John Fitch, Elaine Lary, Sarah Fitch, Melissa SoRelle, Aval-Na'Ree Green

**Affiliations:** ^1^Birkeland Current, Waco, TX, United States; ^2^Division of Geriatric Medicine, Department of Medicine, Baylor Scott & White Medical Center, Temple, TX, United States

**Keywords:** recruitment, Alzheimer's disease, dementia, COVID-19, caregiver, dyad, cost, community-based

## Abstract

Under ideal circumstances, recruitment of older adult-caregiver dyads to dementia research is challenging. The COVID-19 pandemic introduced additional barriers to recruitment, necessitating swift adjustments to pre-pandemic recruitment strategies and schedules. This brief research report describes the challenges, yield, and cost of recruiting older adult-caregiver dyads to an 18-month observational research study during COVID-19. The study aimed to evaluate the effectiveness of a novel in-home sensor system at identifying cognitive change in older adults with Alzheimer's disease and related dementias (ADRD) through background monitoring of activities of daily living (ADL). Recruitment methods included flyers distributed alongside home-delivered meals, direct mailings, publicly available brochures, community presentations, periodical advertisements, and various other strategies. Of 510 inquiries, 117 older adult-caregiver dyads were enrolled at a total cost of ~$368,000, yielding an average per dyad recruiting cost of $3,148. Distributing flyers alongside home-delivered meals produced the most dyads (*n* = 46, 39%) and the least non-labor costs ($24.33) per enrolled dyad. Recruitment during the pandemic exceeded the pre-COVID-19 budget, but enrollment goals were nevertheless achieved through community-based methods. Our experience illustrates the challenge of recruiting older adult-caregiver dyads to dementia research and the value of trusted community partners in recruiting this population. Our strategies and recommendations may benefit researchers who plan to recruit community-based older adults and their caregivers for future dementia research.

## 1. Introduction

Recruitment for any research study is a complex endeavor that can prove challenging, but recruitment of persons with dementia is notably difficult (Grill and Galvin, 2014; Watson et al., 2014; Fargo et al., 2016; Nuño et al., 2017; Bartlett et al., 2019). Recruitment of older adult-caregiver dyads adds another layer of difficulty because both parties must be eligible and consent to participate (Nahm et al., 2012; Field et al., 2019). The COVID-19 pandemic only amplified existing recruitment challenges and introduced novel obstacles to enrolling a representative sample. Lockdowns, social distancing measures, and heightened health concerns disrupted many carefully laid pre-pandemic recruitment plans. With COVID-19 having drastically reshaped the recruitment landscape, understanding which recruitment methods proved successful for recruiting persons with dementia and their caregivers during the pandemic offers insight into the planning and execution of future human-subjects research.

This brief research report presents our experience with recruiting older adult-caregiver dyads to an Alzheimer's disease and related dementias (ADRD) observational research study to validate a novel in-home sensor technology amid the COVID-19 pandemic. The nature of the study as it concerned ADRD, dyads, and technology meant that recruitment was expected to be demanding, but COVID-19 only intensified the demand by necessitating a complete overhaul of the original recruitment strategy. In this paper, we aim to (1) describe the revised community-based recruitment plan, (2) examine the effectiveness of each community-based recruitment method based on cost and participant yield, (3) evaluate the overall impact of COVID-19 on recruitment, and (4) provide lessons learned regarding the recruitment of older adult-caregiver dyads for future ADRD research.

## 2. Method

### 2.1. Study overview

In April 2020, Birkeland Current, along with Baylor Scott & White Health, the Georgia Institute of Technology, and the Texas A&M Center for Population Health and Aging were awarded the Small Business Innovative Research (SBIR) Phase II program R44AG065118 for *Improved AD/ADRD Assessment Sensitivities Using a Novel In-Situ Sensor System*. The purpose of the study was to validate a new activities of daily living (ADL) monitoring system to assess cognitive decline in ADRD adults ages 65 and over. Using radio frequency identification (RFID) technology, the system continuously tracks device usage and real-time location to identify behavioral patterns (e.g., ADL) and objective changes to those patterns associated with physical or cognitive decline. System-derived ADL scores were compared to scores recorded from monthly telephone surveys with caregiver informants using the Alzheimer's Disease Cooperative Study—Activities of Daily Living (ADCS-ADL) Scale (Galasko et al., 1997). The recruitment goal included 108 older adult-caregiver dyads in home settings (i.e., single-family homes, apartments, independent living communities) and 32 dyads from assisted living facilities. The sensor system was to be installed in the residences of older adult participants for an intended 18 months. Eligibility requirements for older adults—henceforth referred to as care recipients (CRs)—and caregivers (CGs) can be found in the Supplementary material. A full overview of the ADL monitoring system is available at sovrinti.com. All elements of the original and updated study protocols were managed and approved under the Texas A&M University institutional review board (IRB) process.

### 2.2. Original recruitment approach and budget

The pre-COVID-19 recruitment plan relied upon commitment letters from nine assisted living, home care, and home health companies representing a total client base of over 3,000 older adults. These care companies committed to recruiting study participants using materials and training provided by the researchers. From discussions with these strategic partners, approximately 900 clients were expected to meet the study's inclusion criteria for CRs and included the expectation that the majority of CG participants would be professional CGs employed by the various companies. CRs and CGs were each to be paid $75 per month as a participation incentive which was classified separately from the recruitment budget. $212,503 was budgeted for direct recruitment costs, including $300 for materials, $6,602 for travel (i.e., mileage reimbursement), and $205,601 for labor not including indirect costs or fringe benefits. The travel recruitment budget was intended to cover two trips to participants' homes—one consent appointment and a subsequent visit to install the sensor system. While the care companies were to perform the bulk of educating and recruiting participants, the labor budget was to cover two research staff members' presence at consent appointments and subsequent sensor system installation appointments. The recruitment plan anticipated a 4-month consent and sensor installation schedule, with an additional 4-month available margin. Based on care company partner populations, recruitment efforts were expected to be concentrated within a narrow geographic area surrounding the Birkeland Current facilities in Waco, Texas. An average of 60 miles round trip per CR household was used to estimate labor and mileage calculations for recruitment. Informed by the demographics of Central Texas, the resulting CR sample was projected to be 67% female, 33% male, 80% white, 20% Black or African American, 70% not Hispanic or Latino, and 30% Hispanic or Latino. The CG sample was projected to be 85% female, 15% male, 70% white, 30% Black or African American, 76% not Hispanic or Latino, and 24% Hispanic or Latino.

### 2.3. COVID-19 impacts and revised recruitment approach

On April 15, 2020, the NIA approved the Phase II program to begin recruitment starting May 1, 2020. On April 30, 2020, Texas A&M halted all human subjects research under their purview until appropriate protocols for accomplishing research during COVID-19 could be established. Birkeland Current worked with the Texas A&M Office of Sponsored Research to establish additional screening and safety protocols to allow the research to proceed beginning in August 2020. Between April and August 2020, all nine care partner companies formally or informally suspended their support for the study. Reasons cited by the companies for suspension included: (1) policy changes restricting non-essential personnel's access to CR's residences; (2) dramatic reductions in client bases due to COVID-19 fears, lockdown policies, or family-imposed restrictions; (3) significantly elevated CG turnover and reductions; and (4) a need to focus on existential business concerns that did not include research support.

The COVID-induced withdrawal of the nine companies' support necessitated a complete overhaul of the original recruitment plan and protocols. In the absence of care company partners to act as intermediaries between the research team and potential participants, all recruitment efforts shifted to the recruitment team at Birkeland Current. Recruitment pivoted to a community-based approach that included the following: flyers distributed alongside home-delivered meal programs (e.g., Meals on Wheels), direct mailings, publicly available brochures, community presentations (both in person and remote), event booths (both in person and remote), Facebook advertisements, magazine advertisements, newsletter articles, new partnerships with assisted living facilities (ALFs), newspaper advertisements and articles, press releases, radio public service announcements, referrals from medical professionals, website postings, and word of mouth. These updated recruitment methods and documents received IRB approval in September 2020.

To facilitate the distribution of IRB-approved flyers alongside home-delivered meals, recruitment staff emailed and called approximately 26 home-delivered meal programs administered by groups such as Meals on Wheels, local senior centers, and other non-profit organizations. Recruitment staff educated meal program administrators on the research study and requested a one-time distribution of study flyers alongside home-delivered meals. Seventeen of the 26 programs agreed to deliver flyers to their client bases. Direct mailings included an IRB-approved postcard sent to potentially viable research candidates. Mailing lists were purchased to target different populations: zip codes within 150 miles of Waco, Texas, adults ages 65 and older, and adult children (ages 45 to 65) that might be acting as CGs to aging relatives. Publicly available or displayed brochures included those delivered to senior centers, senior apartments, medical offices, churches, pharmacies, and community recreation centers. Recruitment staff contacted representatives from these sites and requested to provide brochures for patrons to take. A full overview of recruitment methods can be found in Table 1.

**Table 1:** Recruitment methods.

**Recruitment methods**	**Description**
Direct mailings	Staff purchased targeted mailing lists and sent postcards to potentially viable research participants
Publicly available brochures	Staff contacted senior centers, senior apartments, medical offices, churches, and community recreation centers to request that study brochures be made available in common areas for patrons to take
Community presentations	Staff made presentations both in person and virtually at CG support groups, senior centers, retirement communities, CG agency lunch and learns, and social clubs. Presentation attendees were shown a video describing the study and given informational brochures to keep and distribute to other potentially eligible individuals
Event booths	Staff offered study recruitment materials at an Alzheimer's Association Walk to End Alzheimer's, a local farmer's market, and a regional medical center lobby
Facebook advertisements	Facebook advertisements featuring a video introducing the study targeted adult children that might be caring for aging parents
Magazine advertisements	One advertisement was placed in the official magazine of a 55+ community with over 15,000 residents
Newsletter articles	Announcements about the study were placed in electronic newsletters disseminated by aging resource agencies, caregiving agencies, adult day care centers, and churches
New partnerships with assisted living facilities (ALFs)	Staff emailed and called ALFs to solicit new partnerships whereby the sensor system would be installed in the entire facility. Staff educated ALFs on the study before subsequently making presentations to residents at the facilities that agreed to serve as facility partners
Newspaper advertisements and articles	2-3-week study advertisements were placed in primarily rural newspapers. Staff also contacted rural newspapers and explained the research. If newspaper staff were receptive, the recruitment team subsequently submitted a 300-word article about the study for printing
Press releases	Press releases were submitted to professional board listings
Radio public service announcements (PSAs)	Staff contacted the local radio station and requested they run PSAs regarding the study
Referrals from medical professionals	General practitioners, neurologists, and gerontologists were recruited to disseminate information and flyers regarding the study
Website postings	Information about the study was available on ClinicalTrials.gov and Alzheimers.gov
Word of mouth	The research team encouraged prospective and enrolled participants to share about the study with family and friends. Enrolled participants were given additional study flyers/brochures to share with others

### 2.4. Recruitment flow

From September 2020 to December 2021, CR-CG dyads were recruited to the study via the above methods. Recruitment utilized a two-step screening process whereby interested dyads were first screened over the phone or in person using a brief script. If the dyad met the initial eligibility criteria, an in-home consent meeting was scheduled. At the consent appointment, two researchers educated dyads on the study and administered the Mini-Mental State Exam (MMSE) (Folstein et al., 1975) to potential CRs to assess cognitive function. The MMSE was also administered to CGs over 75 years old. CRs with MMSE scores of 11–25 and CGs with scores of 25 and above were deemed eligible for study participation. Eligible dyads could then consent to the study or decline to participate. At the consent meeting, researchers also measured the layout of CRs' homes to prepare for the sensor system's installation. An appointment was then scheduled where researchers returned to the CR's home and installed the ADL sensor system.

Recruitment data were captured using an SQL-based, internally developed Customer Relationship Management (CRM) system. This system was fully encrypted for the protection of personal identifiable information (PII). Persons inquiring about the study were classified as either potential Care Recipients, Caregivers, Family Members, or Household Members. The following information was collected from individuals who completed the initial screening process: name, name of potential dyad partner, referral source (i.e., how they heard about the study), date of birth, education level, physical address, phone number, email address, estimated time spent with dyad partner per week, willingness to carry an RFID tag for at least 50% of waking hours, COVID-19 screening questions and vaccination status for individuals screened after February 2021. The following information specific to CRs was also recorded: living situation (i.e., lives alone or with others), mobility status (e.g., mobile, mobile with cane), number of people in the household, number of prescription medications taken, and presence of hospice care. CR-CG dyads that passed the initial screen and participated in consent meetings were asked to complete demographics questionnaires which included additional data regarding race, ethnicity, and gender which was then recorded in the CRM.

## 3. Results

### 3.1. Yield per recruitment method

Table 2 shows the number of inquiries and subsequently enrolled CR-CG dyads resulting from each recruitment method. Between September 2020 and December 2021, recruitment staff received approximately 510 inquiries regarding the study through the various recruitment methods. Flyers distributed alongside home-delivered meals produced the most inquiries and enrollees. Seventeen agencies delivered ~5,863 flyers to clients across 32 counties of Central and Southeast Texas. From these 5,863 flyers, 239 individuals inquired about the study, resulting in the enrollment of 46 dyads. This recruitment method yielded 39% (*n* = 46/117) of dyad participants. Direct mailing produced the second-highest number of inquiries and the third-highest number of enrollees. Of 16,801 mailings, 62 individuals inquired about the study, resulting in 11 dyads enrolled. Direct mailings accounted for 9% (*n* = 11/117) of participating dyads. Word of mouth yielded the second-highest number of enrolled dyads (*n* = 15), accounting for 12.8% of participants.

**Table 2:** Participant yield and cost per recruitment method.

**Recruitment method**	**Number**	**Inquiries**	**Enrolled dyads**	Percentage of enrolled dyads (*n* = 117)^*^	**Cost of materials**	**Cost per dyad**
Flyers alongside home-delivered meals	5,863	239	46	39.3%	$1,119.00	$24.33
Word of mouth	NA	42	15	12.8%	$0.00	$0.00
Direct mailings (i.e., postcards)	16,801	62	11	9.4%	$9,476.00	$861.45
Community presentations	20	53	10	8.5%	$0.00	$0.00
Newspaper advertisements/ articles	10	22	10	8.5%	$751.00	$75.10
Partner ALFs	2	12	10	8.5%	$0.00	$0.00
Publicly available brochures	5,600	17	8	6.8%	$2,120.00	$265.00
Newsletter articles	5	6	3	2.6%	$0.00	$0.00
Referrals from medical professionals	NA	2	2	1.7%	$0.00	$0.00
Website listings (clinicaltrials.gov; alzheimers.gov)	2	4	2	1.7%	$0.00	$0.00
Event booths	4	7	0	-	$6,000.00	-
Facebook advertisements	5	1	0	-	$104.99	-
Magazine advertisement	1	0	0	-	$300.00	-
Press releases	3	0	0	-	$0.00	-
Radio public service announcements	2	0	0	-	$0.00	-
Unknown/Not reported	NA	43	0	-	-	-
Total		510	117	-	$19,870.99	$169.84

* Percentages do not total 100 due to rounding.

### 3.2. Total cost and cost by recruitment method

The total expense for recruitment was $368,315.82, for an average cost of $3,148 per enrolled dyad. Of the total, labor accounted for $322,208.83, travel expenses (i.e., mileage reimbursement, gas, meals) accounted for $26,236, and materials accounted for $19,870.99. Based on non-labor costs, direct mailings were the most expensive recruitment method, costing $9,476 and yielding 11 dyads for an average cost of $861.45 per dyad (Table 2). Similarly, from a non-labor cost perspective, flyers distributed alongside home-delivered meals proved the most cost-effective, costing $1,119, representing an average cost of $24.33 per enrolled dyad. Event booths were the least cost-effective, costing $6,000 and yielding no enrollees. Personnel hours associated with individual recruitment methods were not recorded.

### 3.3. Recruitment flow and resulting sample demographics

**Figure 1:**
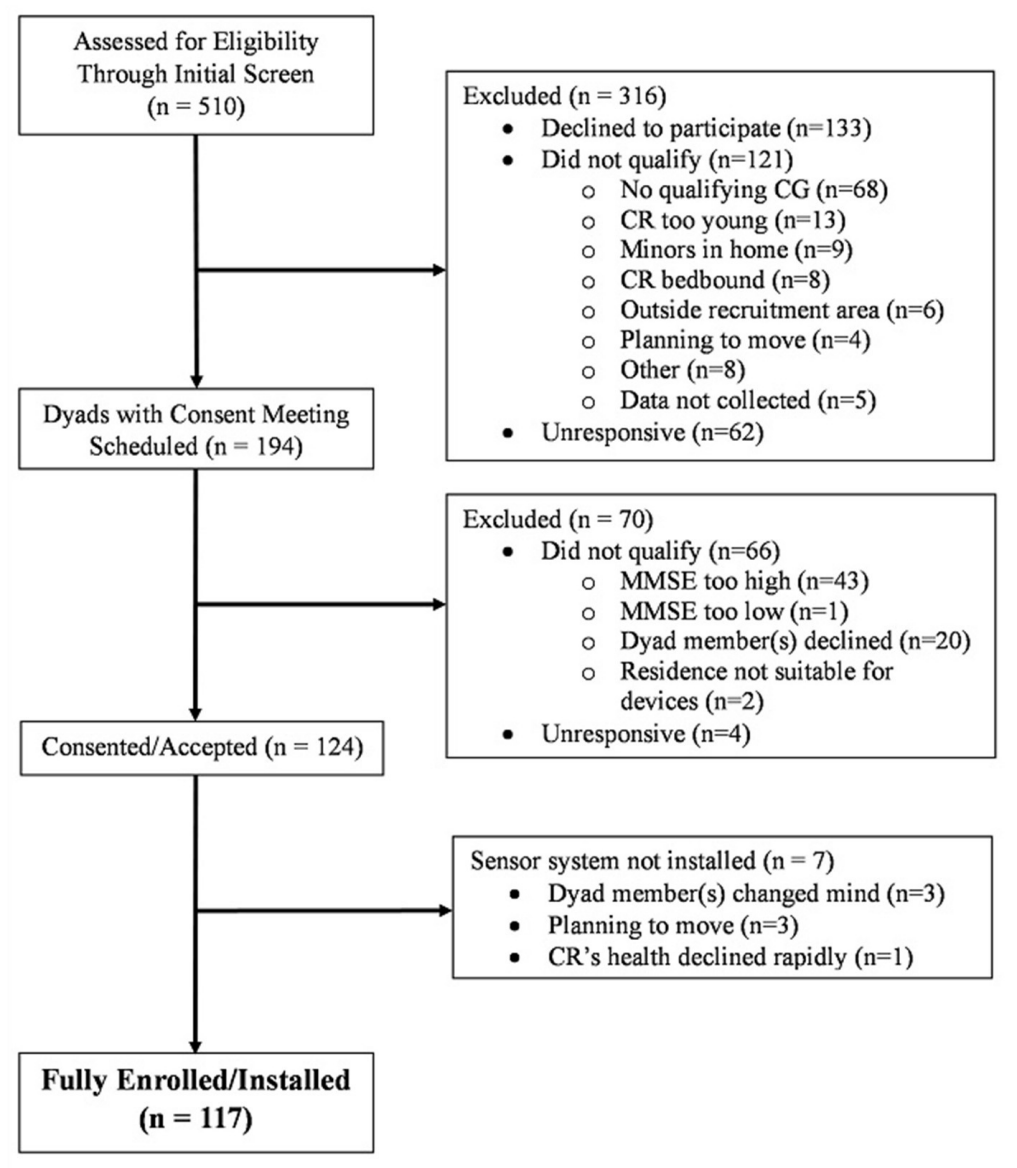
Flow diagram of dyad recruitment.

Of the 510 inquiries yielded by all recruitment methods, 117 CR-CG dyads were fully enrolled (i.e., consented and installed) into the study, with 107 dyads representing CRs living at home and 10 representing CRs living in ALFs. Figure 1 presents a flowchart depicting the outcome of the two-step eligibility screening for the study. Of the initial 510 inquiries, 316 (62%) individuals were excluded during the initial screening step. The primary reason for exclusion was that the individual declined to participate (*n* = 133). Subsequent consent meetings were scheduled with 194 dyads, with 70 dyads being deemed ineligible for the study. The primary reason for ineligibility was that the CR scored too high on the MMSE to qualify (*n* = 45). Of 124 dyads accepted into the study, 7 withdrew consent before the installation of the sensor system into the CR's residence, resulting in the installation of 117 dyads. The average distance traveled per CR home installed was 168 miles roundtrip for 2 in-home visits including the consent meeting and the subsequent installation of the sensor system.

Table 3 shows the demographic characteristics of CRs and CGs fully enrolled in the study through all recruitment methods. As CG turnover was allowed during the study, the demographic data reflects all CGs enrolled from the start of recruitment until study completion (*n* = 123). The majority of CRs were female (*n* = 65; 56%), White (*n* = 81; 69%), and had at least some college education (*n* = 79; 68%). The mean CR age was 79.4 (SD =8.3) years. The majority of CGs were female (*n* = 94; 76%), White (*n* = 87; 71%), and had a mean age of 60.6 (SD=15.3). Spouses accounted for the largest portion of CGs (*n* = 41; 33%) followed by professional CGs (*n* = 39; 31%) and adult children (*n* = 25; 20%).

**Table 3:** Participant demographic information (*N* = 240).

**Characteristic**	**Care recipients (*N* = 117)**	**Caregivers (*N* = 123)**
Age in years, mean (SD)	79.3 (8.3)	60.6 (15.3)
MMSE, mean (SD)	22.6 (2.7)	27.8 (1.5)^*^
**Gender**, ***n*** **(%)**		
Female	65 (55.6)	94 (76.4)
Male	50 (42.7)	27 (22)
Other	0 (0)	1 (0.8)
Not reported	2 (1.7)	1 (0.8)
**Race**, ***n*** **(%)**		
American Indian	1 (0.9)	0 (0)
Asian	0 (0)	1 (0.8)
Black or African American	23 (19.7)	25 (20.3)
White	81 (69.2)	87 (70.7)
Other	6 (5.1)	4 (3.3)
Prefer not to answer	4 (3.4)	5 (4.1)
Not reported	2 (1.7)	1 (0.8)
**Ethnicity**, ***n*** **(%)**		
Hispanic/Latino	6 (5.1)	11 (8.9)
Not Hispanic/Latino	94 (80.3)	99 (80.5)
Prefer not to answer	14 (12)	10 (8.1)
Not reported	3 (2.6)	3 (2.4)
**Education**, ***n*** **(%)**		
Less than a high school degree	16 (13.7)	5 (4.1)
High school degree	18 (15.4)	33 (26.8)
Some college	29 (24.8)	34 (27.6)
Associate degree	9 (7.7)	13 (10.6)
Bachelor's degree	25 (21.4)	26 (21.1)
Master's degree	13 (11.1)	10 (8.1)
Professional degree	3 (2.6)	2 (1.6)
Prefer not to answer	1 (0.9)	0 (0)
No answer	3 (2.6)	0 (0)
**Care recipient living alone**, ***n*** **(%)**		
Yes	38 (32.5)	
No	79 (67.5)	
**Caregiver relationship to care recipient**, ***n*** **(%)**		
Adult child		25 (20.3)
Hired (i.e., formal) caregiver		39 (31.7)
Spouse		41 (33.3)
Other Family		8 (6.5)
Other (i.e., roommate, friend, volunteer, etc.)		10 (8.1)

* N = 28 CGs aged 75 years and over with whom an MMSE was completed.

## 4. Discussion

This paper presents a narrative of COVID-19's impact on the recruitment of CR-CG dyads for an observational ADRD study and the outcomes of community-based recruiting. COVID-19 drastically shaped this study's recruitment process. Due to COVID-related concerns, the nine care companies that were to serve as recruitment associates suspended their partnership. This necessitated a swift pivot to a community-based recruitment approach where all recruitment responsibilities (i.e., outreach, initial screen, cognitive evaluation/MMSE, and consent) shifted to the Birkeland Current research team, leading to greater labor, travel, and materials costs as well as a different sample composition than anticipated. Despite the challenges introduced by COVID, the original recruitment goal was largely met through various community recruitment methods, providing insights for future researchers regarding the effectiveness and costs of strategies.

In our experience, home-delivered meal programs' willingness to distribute flyers proved invaluable to recruiting CR-CG dyads for an ADRD study during COVID. Unlike many medical practices, care companies, and social service agencies, home-delivered meal programs largely retained access to their clients during COVID, providing meals to a population that became isolated to an even greater extent during the pandemic (Lebrasseur et al., 2021). As a trusted service provider with access to a sizeable, diverse population, home-delivered meal programs were well-positioned to disseminate study flyers alongside meals even during a pandemic. Once they understood the research and the meaningful impact of their assistance, most leaders in these organizations graciously offered their support. The research team also received feedback from organizations that including study flyers with meals did not add an undue burden to their operations. Reasons cited by home-delivered meal programs for not distributing flyers included: (1) present operations too strained by COVID; (2) currently prioritizing information about COVID-relief resources; (3) already working with other researchers to disseminate recruitment materials and do not want to inundate clients; (4) privacy concerns related to in-home sensor technology.

Flyers distributed alongside home-delivered meals ultimately generated the most inquiries and the highest number of enrolled dyads for the least cost among recruitment methods with associated expenses for materials. The better response rate obtained via flyers disseminated by home-delivered meal programs (4.1%) compared to the response rate of direct mailings (.4%) is consistent with the finding that older adults are more likely to respond to surveys provided by someone known to them than surveys sent in the mail (Edelman et al., 2013). Results suggest that distributing flyers alongside home-delivered meals represents a promising recruitment strategy for recruiting CR-CG dyads for ADRD research.

Newspaper advertisements and articles proved to be another relatively efficient and cost-effective recruitment strategy, with 10 ads/articles yielding 10 dyads representing an average cost of $75.10 per dyad based on materials. Submitting ads and articles to newspapers required less time compared to other labor-intensive methods like presentations and soliciting new partnerships with ALFs.

Word of mouth emerging as the second-highest source of dyad enrollment came as a surprise to the research team. While researchers had encouraged prospective and enrolled participants to share about the study with family and friends, word of mouth was seen as a secondary recruitment method involving a less systematic, concerted effort such as might be done in snowball sampling. As a low-cost, relatively low-effort recruitment method, word of mouth proved to be a valuable component of our community-based recruitment effort. These results suggest the efficacy of word-of-mouth in recruiting participants to ADRD research which is in keeping with historical uses of snowball sampling to recruit hard-to-reach populations (Heckathorn, 2011).

In contrast to flyers, newspaper ads/articles, and word of mouth, targeted mailings, event booths, and social media proved inefficient in our experience. While direct mailings yielded 9% of enrolled dyads, it was the costliest method in terms of materials (including contact list costs and postage) and resulted in the highest average non-labor cost per dyad. Event booths were also expensive while yielding no enrollees. Although relatively low-cost, Facebook ads generated only one inquiry and no dyads.

Overall, community-based recruitment cost considerably more than what was originally budgeted based on the pre-COVID-19 care company partnership approach. Labor costs exceed the original budget by 64% as recruitment tasks shifted to the research team and necessitated additional staff (i.e., four paid interns). That community-based recruitment took longer than the care company partnership was expected to take also contributed to the higher recruitment cost. While care company partners would have recruited from their existing client bases that mostly met initial eligibility criteria surrounding age and availability of a CG partner, community-based recruiting involved the additional step of having to first identify potential participants who met these inclusion criteria and subsequently determine if they qualified for the study. This added step in recruitment led to a longer recruitment process which impacted labor costs. Similarly, travel constituted a greater expense due to having to recruit participants from beyond the originally targeted geographic zone to meet recruitment goals. The recruitment zone was expanded to include Dallas, Austin, and Houston, each situated at distances ~200 miles, 340 miles, and 200 miles roundtrip from the research offices in Waco, respectively. While the original recruitment plan anticipated that participants would be clustered in geographic areas served by the nine care company partners, the sample resulting from community-based recruitment was more dispersed. Consequently, the distance traveled to the 117 CR households exceeded original projections by 180%, averaging 168 miles per household compared to the estimated 60 miles per household. Additionally, materials cost 6,524% more than originally budgeted. This is because the planned recruitment approach only necessitated flyers and brochures for the nine care company partners to distribute. The COVID-induced pivot to community-based recruitment methods required substantially more materials, leading to a significantly higher cost.

In addition to its impact on cost, the COVID-induced shift in recruitment approach also yielded a different sample than anticipated. While community-based methods yielded nearly the desired number of CRs living at home (107 CRs compared to the targeted 108), we did not meet the recruitment goal for CRs in ALFs, enrolling only 10 CRs in ALFs compared to the desired 32. This outcome was unsurprising given the withdrawal of the original ALF strategic partners and the difficulty of forging new partnerships with ALFs during COVID-19 when even family members were often not allowed access to facilities. The original recruitment plan also assumed the majority of CG participants would be formal CGs employed by the nine care company partners. In contrast, community-based recruitment resulted in informal CGs (i.e., spouse, adult child, other family, friend, etc.) constituting 68% of CG participants. Based on initial screen reports, this majority informal CG population spent more time with CRs per week (35 h on average) than the formal CGs (24 h on average), with 77% of informal CGs living with their CR dyad partner. With CGs serving as the primary informants of CR ADL performance via monthly surveys, we expect this unanticipated majority informal, live-in CG sample to shape future data analyses and interpretation of CG survey results.

Compared to original projections, the enrolled CR sample consisted of more men than anticipated. While we met the target of enrolling a CR sample that was at least 20% Black/African American, we fell below the anticipated percentage of Hispanic/Latino participants, recruiting a sample only 5% Hispanic/Latino compared to the expected 30%. We similarly yielded fewer Black/African American and Hispanic/Latino CGs than estimated. These outcomes are consistent with literature noting the underrepresentation of racial and ethnic minority populations in ADRD research (Olin et al., 2002; Areán et al., 2003; Gilmore-Bykovskyi et al., 2019). Also, study inclusion criteria required participants to be English speakers, which potentially created a barrier to recruitment. Similarly, recruitment methods targeting minority populations were not employed, which may have contributed to a less diverse sample.

### 4.1. Insights

Based on our experience of recruiting CR-CG dyads for an ADRD research study during the COVID-19 pandemic, we offer several preliminary insights and recommendations for future researchers. First, the response to flyers distributed alongside home-delivered meals highlights the encouraging prospects of employing this cost-effective method for recruiting CR-CG dyads for ADRD research. We encourage researchers to engage with home-delivered meal programs and other trusted community organizations to aid in recruitment. While many organizations may not have the margins to actively recruit or refer research participants, distributing flyers to clients represents a less labor-intensive alternative. Secondly, the resulting sample of majority informal CGs illustrates the impact that recruitment methods have on sample demographics. We anticipated recruiting more formal CGs via care company partners, but the pivot to community-based methods yielded a majority informal CG sample. If researchers seek a majority formal CG population, community-based recruitment methods may not be most effective. Thirdly, the shift in recruitment approach dictated by COVID-19 and the resulting costs showcase the need for researchers to be flexible and mindful of the time and expense needed to recruit CR-CG dyads for ADRD research. Community-based recruitment required more concerted effort than expected, which necessitated staffing and budgetary adjustments. While COVID-related complications could not have been predicted, we might have anticipated potentially needing alternative recruitment strategies to supplement the originally planned care company partnership approach.

### 4.2. Limitations

There are limitations in obtaining and applying insights based on this recruitment narrative. First, CR eligibility was based on unadjusted MMSE scores, resulting in more CRs being accepted than if eligibility required an official ADRD diagnosis or was based on MMSE scores adjusted for age and education. Second, the monthly CG surveys were only conducted in English, resulting in a less linguistically and ethnically diverse CG sample. Third, participants were compensated for their involvement in the study, which may have inclined them to participate. Fourth, we did not record the time and associated labor costs associated with individual recruitment methods, which would need to be explored to fully assess the cost-effectiveness of different recruitment strategies. Fifth, the recruitment source was unknown for 8% (*n* = 43) of inquiries. These represent inquiries that could not be followed up on as well as people who declined to participate before information about the recruitment source could be collected. Had the information been collected, it may have impacted which recruitment methods were considered most successful and cost-effective. Lastly, we cannot ascertain whether individuals who inquired based on one recruitment method would have inquired in response to another strategy.

### 4.3. Summary

Despite the multi-layered challenges associated with recruiting CR-CG dyads for an ADRD research study involving in-home sensor technology during the COVID-19 pandemic, our results demonstrate the feasibility of using community-based recruitment methods—specifically flyers distributed alongside home-delivered meals—to enroll participants. We anticipate that our transparency regarding recruitment challenges and costs will aid future researchers in planning for the successful recruitment of participants to ADRD studies.

## Data availability statement

The raw data supporting the conclusions of this article will be made available by the authors, without undue reservation.

## Ethics statement

The studies involving humans were approved by Texas A&M University Institutional Review Board IRB00000397. The studies were conducted in accordance with the local legislation and institutional requirements. The participants provided their written informed consent to participate in this study.

## Author contributions

RW: Data curation, Formal analysis, Investigation, Visualization, Writing—original draft. JF: Conceptualization, Funding acquisition, Investigation, Methodology, Project administration, Resources, Supervision, Writing—review and editing. EL: Data curation, Formal analysis, Investigation, Project administration, Resources, Supervision, Writing—review and editing. SF: Investigation, Writing—review and editing. MS: Investigation, Writing—review and editing. A-NG: Conceptualization, Supervision, Writing—review and editing.
