# Supplementary material for: Recruitment of older adult-caregiver dyads during the COVID-19 pandemic: an example from a study to evaluate a novel activities of daily living (ADL) sensor system
Source: Front Dement. 2023 Oct 19;2:1271754. doi: 10.3389/frdem.2023.1271754 (PMC11285568; doi:10.3389/frdem.2023.1271754)
Supplement: Supplementary file 1 [file Table_1.docx]

Supplementary Material

Recruitment of Older Adult-Caregiver Dyads During the COVID-19 Pandemic: An Example from a Study to Evaluate a Novel Activities of Daily Living Sensor System

Rachel Williams*, John Fitch, Elaine Lary, Sarah Fitch, Melissa SoRelle, Aval-Na’Ree Green

*** Correspondence:** Rachel Williams: rachel.williams@birkelandcurrent.com

# Supplementary Tables

**Supplementary Table 1. Population A.1 Care Recipient Eligibility Criteria**

| **Eligibility Criteria** |
| --- |
| Must be fluent in English to understand and sign the consent or assent documents. |
| Must be an adult >65 years old living in a community-dwelling residence with no children under 18 years old living in the residence. |
| Adult anticipates he/she will be living primarily at designated home or assisted living facility (ALF) over next 18 months (i.e., not planning to move and does not expect to be traveling away from home or ALF more than 30 days over the next 18 months). |
| Must receive caregiver interaction at least 6 hours/week. |
| Must undergo a Mini-Mental State Exam (MMSE) assessment at baseline during recruitment as well as at the end of the study with a resulting score of 11-25 as part of the recruitment phase of the study. |
| Must be willing to have home or facility equipped with sensors to obtain activity and location data. |
| Must be willing to wear an active Radio-Frequency Identification (RFID) badge tag or key fob either on (i.e., clip) or in their apparel (i.e., pocket) while at home for location and activity tracking at least 50% of the time they are awake. |
| Must be willing to undergo an MMSE assessment at the end of their participation. |
| Must consent to participate by signing the consent “Study_2_IC_CR_v3_10” or assent “Study_2_IA_CR_v1_2.” |

**Supplementary Table 2. Population B.1 Caregiver Eligibility Criteria**

| **Eligibility Criteria** |
| --- |
| Must be fluent in English to understand and sign the consent or assent documents. |
| Must have a high school diploma or equivalent education to understand the consent document. |
| Must be an adult at least 18 years old providing care to A.1 participant at least 6 hours per week. |
| For B.1 participants over 75 years old, must complete an MMSE with a score of >25. |
| Must agree to answer an assessment questionnaire monthly about the activities of a Population A.1 care recipient. |
| Must agree to answer a survey questionnaire at baseline and at 9- and 18-month follow-up from the start time of the A.1 participant about their caregiving activities and attitudes and beliefs about technology and caregiving. |
| Must be planning to continue providing care to an A.1 participant for 18 months if being recruited at the beginning of the study or for the remainder of 18 months from the time the last caregiver exited as a participant. |
| Must not be pregnant. |
| Must be willing to have activity and location data collected while providing care to participating care recipient. |
| Must be willing to wear an active RFID badge tag or key fob either on (i.e., clip) or in their apparel (i.e., pocket) while providing care to participating care recipient for activity and location tracking. If they live with an A.1 participant, they must be willing to wear the tag at least 50% of the time they are awake. |
| Must consent to participate by signing the “Study_2_IC_CG_v2_10” form to participate. |
